# Face validity and reliability test of the Danish version of the compliance questionnaire rheumatology in patients with early rheumatoid arthritis

**DOI:** 10.1186/s41927-023-00364-5

**Published:** 2023-10-25

**Authors:** Line Raunsbæk Knudsen, Annette de Thurah

**Affiliations:** 1 Department of Rheumatology, Aarhus University Hospital, Aarhus, Denmark; 2 Department of Clinical Medicine, Aarhus University, Aarhus, Denmark

**Keywords:** Adherence, Compliance, Rheumatic and Musculoskeletal Diseases, Measurement properties

## Abstract

**Background:**

Supporting adherence to medication is an essential part of the treatment and care of patients with rheumatic and musculoskeletal diseases. The Compliance Questionnaire Rheumatology (CQR) measures adherence in rheumatic diseases through 19 items covering drug-taking behaviour to identify the reasons for adhering to treatment and the factors that contribute to suboptimal adherence. The objective of this study was to present the translation of the CQR into Danish and the face validity and reliability test.

**Methods:**

The CQR was translated into Danish according to international guidelines, followed by a face validity test among 10 patients with rheumatoid arthritis in 2009. The test–retest reliability of the Danish CQR was evaluated in 49 patients with rheumatoid arthritis in 2020 - 2021 using the standard error of the measurement (SEM) converted into the minimally detectable change (MDC) and the intraclass correlation coefficient (ICC). Questionnaires were administered with a minimum of 10 days between assessments.

**Results:**

The participants in the reliability test had a mean age of 57.4 years (SD 16.1) and a mean disease duration of 1.13 years (range 2 months–2 years). The mean CQR score in the test and retest was 62.7 (confidence interval (CI) 58.8; 66.6) and 62.5 (CI 58.9; 66.1), respectively, with a SEM of 8.59 (7.16; 10.73) and an MDC of 16.83. A satisfactory test–retest reliability was confirmed by an ICC value of 0.79 (CI 0.68; 0.89).

**Conclusion:**

The Danish CQR has satisfactory test–retest reliability in patients newly diagnosed with rheumatoid arthritis and is considered a reliable tool to measure adherence in this group.

## Background

Medication adherence in inflammatory arthritis has been reported to vary from 30 to 80%, despite the fact that non-adherence may cause worsening of symptoms and disease severity [1]. According to the European Alliance of Associations for Rheumatology (EULAR), adherence is the behaviour of following a prescription based on shared decision making that allows patient preferences, beliefs and necessities about medication to be considered [1]. As adherence may vary throughout the disease course and may be influenced by several factors, it should also be evaluated continuously throughout the disease course to ensure optimal care and treatment. A trustful relationship and open discussions between patients and healthcare providers is crucial to promote adherence and can be supplemented by, for example, questionnaires to measure adherence [1].

The Compliance Questionnaire Rheumatology (CQR), a validated measure specifically designed to measure drug adherence in patients with rheumatic diseases, showed a sensitivity of 98% and a specificity of 67% to identify non-adherence [2]. It has been evaluated against electronic medication event monitoring in a Dutch study that included 127 patients with rheumatoid arthritis (RA), polymyalgia rheumatica and gout [3]. Through discriminant analyses, this study found a specificity of 95% and a sensitivity of 62% in detecting good taking compliance, and the predictive value was 86% in detecting unsatisfactory taking compliance and 83% in detecting good taking compliance [3].

The CQR was translated into Danish and face-validated among 10 patients with RA in 2009 prior to a study investigating adherence to methotrexate [4]. Therefore, the aim of this article was to present data from the translation, face validity study and reliability test of the Danish CQR.

## Methods

### CQR

The CQR is a self-administered questionnaire [2] consisting of 19 items covering drug-taking behaviour, that is, adherence to treatment, including the reasons to adhere and the identification of factors that contribute to suboptimal adherence. The answer to each question is given on a four-point Likert scale ranging from ‘don’t agree at all’ (scored 1), ‘don’t agree’ (scored 2) and ‘agree’ (scored 3) to ‘agree very much’ (scored 4), with higher scores indicating higher adherence [3]. The CQR total score is calculated and varies from 0 (complete non-compliance) to 100 (perfect compliance) [3].

### Translation and face validity test of the CQR into Danish

**Fig. 1:**
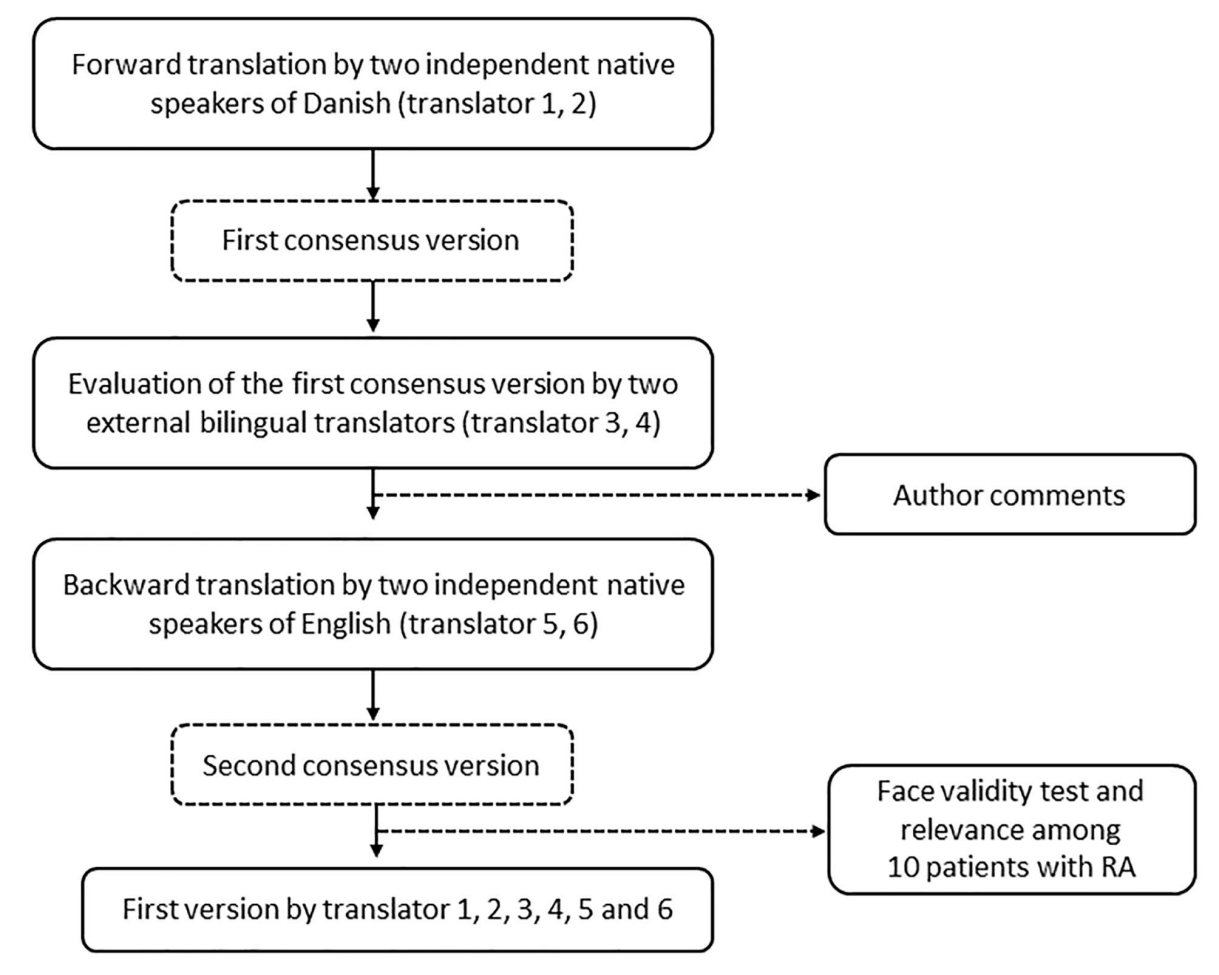
Flow chart of the translation and face validity test of the CQR into Danish Boxes with a solid line shows actions taken in the process, while boxes with a dotted line represents the output of each step

The CQR was translated and face-validated into Danish among patients with RA in a study, which was conducted in 2009 [4]. After the Dutch authors of the original publications gave their approval, the instrument was translated into Danish according to the International Quality of Life Assessment method, which involves forward and backward translations by independent translators and a face validity test [5]. The instrument was tested among 10 patients with RA from the Department of Rheumatology at Aarhus University Hospital for relevance and face validity. The testing was conducted using cognitive interviewing principles, which involved the use of the ‘think aloud’ technique and verbal probing during the interview process [6, 7]. During the test, the patients were asked to express their immediate impressions of the questionnaire, and the single items, and they were instructed to go through the questionnaire and provide feedback along the way, specifically, they were asked about the layout, the response format, relevance and phrasing. Questions such as *‘What comes into your mind when you read this?’, ‘What did you notice when answering this question?‘* or *‘Did you find it easy or difficult to answer this question?‘* were asked during the interview. The feedback from this test did not lead to substantial corrections, as none of the interviewees had problems understanding the questionnaire and all the patients found the content of the questionnaire relevant. Figure 1 presents an overview of the translation and face validity test.

### Reliability test

For the reliability test, adult patients (> 18 years) newly diagnosed with RA (< 2 years) according to the American College of Rheumatology/European League Against Rheumatism 2010 (ACR/EULAR 2010) criteria [8] and with sufficient skills to read and understand Danish were recruited from a rheumatology outpatient clinic at Aarhus University Hospital, Denmark.

Reliability refers to the degree to which the measurement is free from measurement error, that is, the measurement properties: internal consistency, reliability and measurement error [9]. This study used test–retest to evaluate the extent to which the CQR scores changed over time. Thus, the questionnaire was evaluated with a minimum of 10 days between assessments to reduce the risk of the recollection of answers. The questionnaire was sent to the participants at baseline, followed by a new questionnaire (retest) 10 days later. The questionnaires were administered through Research Electronic Data Capture, REDCap, a secure web application for online surveys and databases hosted at Aarhus University [10, 11]. Both questionnaires were completed at home.

## Statistical analysis

Descriptive statistics were calculated for the following variables: age, gender, disease duration, laboratory variables, medical treatment and time between test and retest. Scatters of the differences between the test and retest were plotted against the means to indicate whether the differences were related to the CQR score. The differences between test and retest were calculated, and the systematic differences were assessed using a paired *t*-test. The differences were plotted against the means of the two measurements using Bland–Altman plots, with 95% confidence intervals (CI) and 95% limits of agreement (LOA). Absolute measurement errors were estimated by calculating the standard error of the measurement (SEM) and converted into minimally detectable change (MDC) (MDC = 1.96 × √2 × SEM). The MDC defines the smallest within-person change that can be interpreted as a ‘real’ change above the measurement error [12]. The intraclass correlation coefficient (ICC) model 2.1, with a corresponding 95% CI, was used to assess reliability. The ICC can range from 0.0 to 1.0, and according to recommendations, an ICC exceeding ≥ 0.70 is considered sufficient reliability for the evaluation of individual patients [13]. As the analysis was based on the complete responses to both questionnaires, no missing items were handled. STATA version 17 [14] was used in the statistical analysis.

## Results

**Fig. 2:**
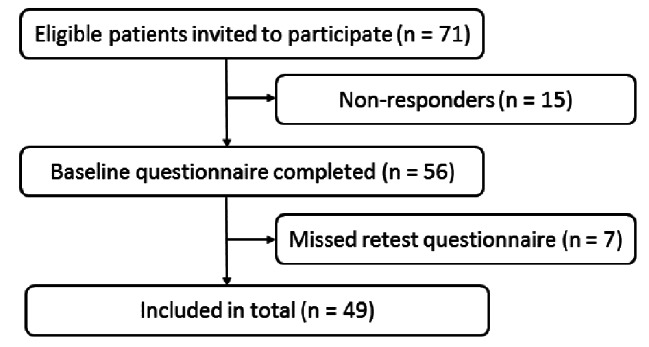
Flowchart for inclusion in reliability test of the Danish CQR

**Fig. 3:**
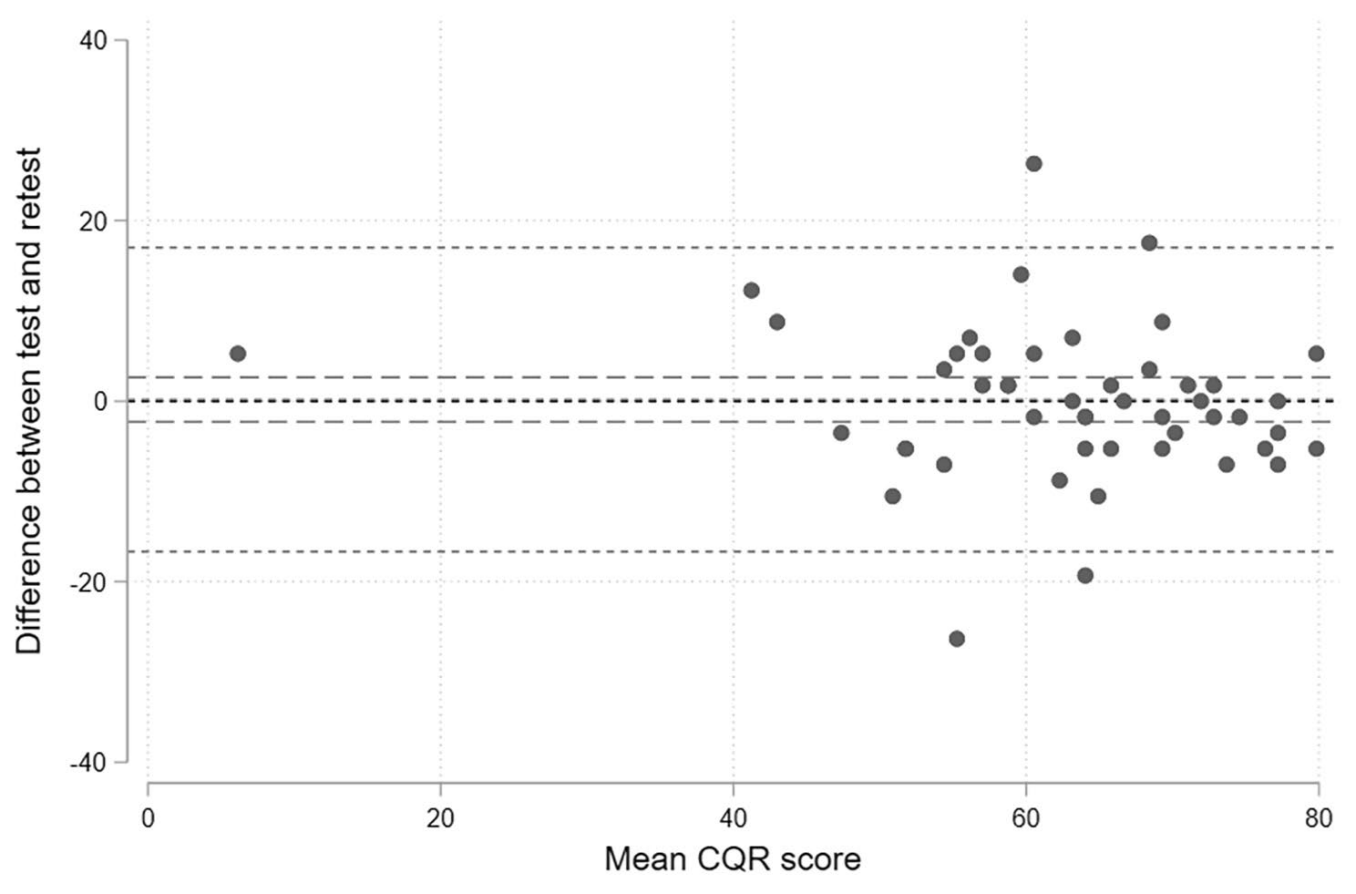
Differences between test and retest plotted against the mean CQR score

Data for the reliability test were collected from October 2020 to March 2021. We invited 71 patients to take part in the study, and of the 56 patients who completed the first test, 49 also completed the questionnaire at retest. Thus, non-responders included 15 patients and 7 patients missed retest. The response rate was 69%. No statistical difference was found between the included patients and the non-responders or patients who missed the retest regarding age, sex, positive rheumatoid factor, positive cyclic citrullinated peptide or methotrexate treatment (*p* > 0.05). Table 1 shows the participant characteristics, and Fig. 2 provides an overview of the inclusion. The mean duration between test and retest was 12.8 days (SD 3.93), and the mean CQR score was 62.69 (58.76; 66.63) by baseline (test) and 62.51 (58.91; 66.12) by retest. Thus, no statistical difference in the mean CQR score was found between test and retest (*p =* 0.88 using paired *t*-test). We found satisfactory test–retest reliability and no systematic bias between measurements, with an ICC value of 0.79 (CI 0.68; 0.89), SEM of 8.59 (7.16; 10.73) and MDC of 16.83 (Table 2). Figure 3 shows a scatter plot of the differences between the test and retest against the mean CQR score.

**Table 1:** Participant characteristics among 49 newly diagnosed patients with RA Values in mean (SD) and [range] or no. and (percentage), as stated

Age in years (mean)	58.26 (16.2)
Sex, female, no. (%)	41 (83.67)
Disease duration in years (SD)	1.12 (0.48)
Rheumatoid factor positive, no. (%)	29 (59.18)
Cyclic citrullinated peptide positive, no. (%)	29 (59.18)
Methotrexate, no. (%)	43 (87.76)
Days between test and retest (SD)	12.8 (3.93) [10–28]

Values in mean (SD) and [range] or no. and (percentage), as stated

**Table 2:** Reliability and agreement parameters for the Compliance Questionnaire Rheumatology (CQR) in 49 patients with rheumatoid arthritis

	Mean (95% CI) test Mean (95% CI) retest	Difference (95% CI)	LOA	SEM (95% CI)	ICC (95% CI)	MDC
CQR19	62.7 (58.8; 66.6) 62.5 (58.9; 66.1)	0.18 (− 2.29; 2.65)	−16.66–17.01	8.59 (7.16; 10.73)	0.79 (0.68; 0.89)	16.83

*LOA* = limits of agreement, *SEM* = standard error of measurements, *MDC* = minimal detectable change, *ICC* = intraclass correlation coefficient model 2.1

## Discussion

We found that the Danish CQR is a reliable tool for measuring adherence among patients newly diagnosed with RA. To strengthen reliability and in accordance with the Consensus-based Standards for the selection of health Measurement Instruments (COSMIN) guidelines [15], we designed a study with two independent measurements and an appropriate time interval of 10 days to prevent recall of answers. Furthermore, the test conditions were similar for the measurements, as both questionnaires were administered electronically and completed at home, without the influence of healthcare providers [15]. According to the guidelines, reliability measured by ICC should exceed ≥ 0.70 as a minimum [13]. We found an ICC of 0.79, which is considered sufficient and indicates a low degree of systematic error in the measurement. The MDC showed that at least 16.83 points were needed to detect a ‘real’ change in the total CQR score.

The limitations of this study could be related to the population. There is a risk that the patients were not stable in the interim period of the measurements, as we included patients newly diagnosed with RA (< 2 years). Thus, disease activity could be more likely to vary within this group, as some could have achieved remission and others could be in high disease activity. Therefore, this instrument is expected to perform better among prevalent cases due to its more stable context. However, we expected a short interval of 10 days between assessments to reduce the risk of disease fluctuations in individuals. Additionally, it should be taken into consideration that there may be differences in adherence between incident and prevalent subjects. Often, adherence tends to be lower among incident users as discontinuation rates are highest in the initial stages of treatment [16]. For example, van den Bemt et al. highlights that patients are more likely to adhere to treatment when they believe it is effective and the benefits outweigh the risks [17]. Patients with early RA commonly experience side-effects of medications such as Methotrexate, and it takes a considerable amount of time before an effect is observed. This could potentially lead to discontinuation or non-adherence to treatment compared to prevalent patients, who have experienced the beneficial effects of the treatment.

The CQR was developed more than 20 years ago and validated against electronic event monitoring as the ‘gold standard’. This has recently been criticised as a one-dimensional validation that leaves no justification for the use of the weighted sum of items. However, the CQR is a user-friendly instrument, frequently used within rheumatology which enables comparison to other studies. Further, we have earlier shown, that high scores of CQR is associated with a high perception of necessity towards the drug, as indicated by the beliefs about medication questionnaire, among RA patients who are incident users of MTX [4]. To some extent, this finding may support the alignment between the CQR and the theoretical construct of adherence.

Another limitation could be that seven patients missed the retest, which reduced the sample from 56 to 49 patients. Although the response rate of both questionnaires was satisfactory (69%) and loss to follow-up was low, the study could have benefited from an extended sample, as the COSMIN guidelines state that 50–99 patients are considered adequate for test–retest reliability [15].

## Conclusion

The Danish CQR has satisfactory test–retest reliability in patients newly diagnosed with RA and is thus considered a reliable tool to measure adherence in this group.

## Data Availability

The Danish version of the CQR, and data on the statistical analysis are available upon request from the corresponding author.

## Abbreviations

ACR: American College of Rheumatology
CQR: Compliance Questionnaire Rheumatology
COSMIN: Consensus-based Standards for the selection of health Measurement Instruments
CI: Confidence interval
EULAR: European Alliance of Associations for Rheumatology
ICC: Intraclass correlation coefficient
LOA: Limits of agreement
MDC: Minimally detectable change
RA: Rheumatoid arthritis
SEM: Standard error of the measurement
SD: Standard deviation
